# Recent extinctions of plant and animal genera are rare, localized, and decelerated

**DOI:** 10.1371/journal.pbio.3003356

**Published:** 2025-09-04

**Authors:** John J. Wiens, Kristen E. Saban

**Affiliations:** 1 Department of Ecology and Evolutionary Biology, University of Arizona, Tucson, Arizona, United States of America; 2 Department of Organismic and Evolutionary Biology, Harvard University, Cambridge, Massachusetts, United States of America; Trent University, CANADA

## Abstract

An important aspect of the current extinction crisis is the loss of distinct clades (e.g., genera). A recent study suggested that there is rapidly accelerating extinction of genera (and other higher taxa), indicating a current mass extinction event that endangers human survival. However, that study was based only on land vertebrates, which include only about half of vertebrates and <2% of living species. Here, we examine the recent extinction (last 500 years) of higher taxa across living organisms. We find that 102 genera have gone extinct (90 animals, 12 plants), along with 10 families and two orders. Yet, the majority of these genus-level extinctions were among mammals and birds, as were all extinctions of families and orders. There were very few extinctions among the thousands of genera of ray-finned fishes (*n* = 4; 0.08%), squamate reptiles (*n* = 2; 0.17%), and amphibians (*n* = 1; 0.18%). Documented extinctions were also rare among the thousands of assessed genera of arthropods (*n* = 11; 0.32%) and plants (*n* = 12; 0.17%), which together encompass most known species. Most extinct genera were monotypic (~80%), and most were island endemics (76%). Moreover, despite the claim that extinctions of higher taxa are rapidly accelerating, the highest rates of genus-level extinctions occurred more than 100 years ago, and have declined subsequently. Overall, the recent extinctions of higher taxa are not as dire as previously suggested.

## Abstract 2

Recent research claims that extinctions of tetrapod genera are rapidly accelerating and threaten human survival. This study shows, through an analysis of recent extinctions, across plant and animal genera, that extinctions were mostly confined to islands, were predominantly of birds and mammals, and have generally declined over the last century.

## Introduction

There is widespread recognition that Earth is experiencing a human-related biodiversity crisis, leading to urgent calls for action [1–3]. One crucial piece of evidence supporting the idea of a biodiversity crisis is that hundreds of species have gone extinct over the last ~500 years because of human impacts [4–6].

Extinctions of genera and other higher taxa may be especially problematic. All else being equal, a distinct genus may represent more evolutionary history than a typical species within a genus, even if that genus is monotypic. They may also be more divergent genetically, morphologically, ecologically, and functionally. Thus, there has been great interest in conservation biology in preserving taxa that are evolutionarily distinct and represent high amounts of phylogenetic history, with higher taxa potentially representing more history than individual species within a genus [7–14]. For example, the extinction of the single species of tuatara (*Sphenodon punctatus*), the sole extant member of a monotypic reptile order (Rhynchocephalia) representing >250 million years of evolutionary history, would potentially be a greater loss than a single species of *Thamnophis*, a snake genus that is ~10 million years old and contains 36 species [15,16]. Higher taxa offer a useful bookkeeping system for recognizing potentially distinct clades, even if these taxa are not equivalent within or between groups (e.g., all genera are not the same age within or between major groups, but then neither are species).

A thought-provoking paper [17] recently highlighted the idea that there have been many recent extinctions of clades ranked above the species level. Those authors [17] used data from the International Union for the Conservation of Nature (IUCN [18]) to document recent extinctions among tetrapod vertebrates (e.g., birds, mammals, amphibians, turtles, lizards, and snakes). They concluded that genus-level extinction rates were “rapidly accelerating.” They also suggested that these extinctions were evidence of a mass extinction event, and that this mass extinction could lead to the widespread loss of ecosystem services and the collapse of human civilization and that it was “destroying the conditions that make human life possible”.

Here, we analyze the extinction of higher taxa across living organisms. Tetrapods encompass <2% of all known species [19], and only about half of known vertebrate species. We address the following questions: (i) how do patterns of extinction of genera (and other higher taxa) vary among major groups of organisms? (ii) How do genus-level extinction rates vary over time? For example, have these rates rapidly accelerated toward the present in tetrapods and in other groups? (iii) How do genus-level extinctions vary over space? Ceballos and Ehrlich [17] suggested that genus-level extinctions were especially prevalent in tropical Asia, Africa, and the Americas. However, other studies have suggested that recent species-level extinctions are especially frequent among island endemics [20–24], even though only ~20% of terrestrial species are thought to occur on islands [21,23]. It remains unclear whether this pattern also holds for extinct genera.

## Results

**Fig 1:**
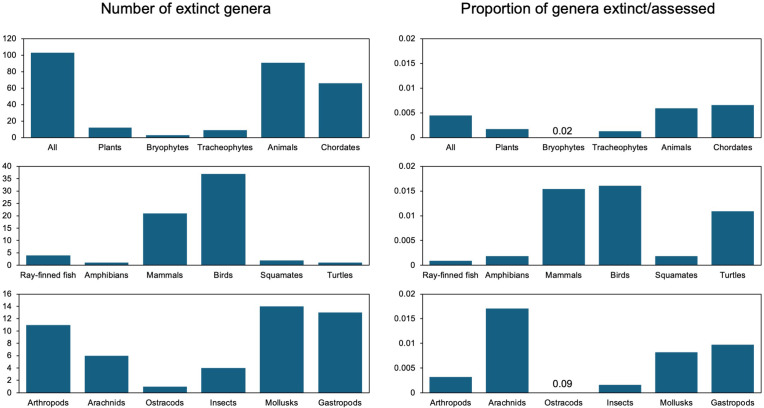
Patterns of recent genus-level extinctions among major groups of animals and plants. On the left-side column, we give the absolute number of genera that have gone extinct in each group (note that y-axes are different in each graph). On the right-side column, we give the proportion of extinct genera among those genera that have had one or more species assessed by IUCN. There were relatively few genera of bryophytes and ostracods assessed, and these had relatively high proportions of extinct genera relative to the number assessed. For these two groups we simply show the number. To conserve space we do not give every result for every group (e.g., bivalves, plant classes). Values for all groups are summarized in S1 Table and data for all genera are given in full in S1 Dataset (available on figshare: https://doi.org/10.6084/m9.figshare.27377613).

We found that 102 genera of plants and animals have gone extinct since 1500 (Fig 1; S1 Table; S1 Dataset; all datasets are available at https://doi.org/10.6084/m9.figshare.27377613). There were no genus-level extinctions documented in other groups (e.g., fungi). Among these 102 genera, 79 were clearly monotypic, whereas 20 had two or more species. For three genera, it was unclear if they had one or two species, but all their species were extinct regardless. These extinct genera encompassed 179 species in total, and the number of species in non-monotypic genera ranged from 2–21 (mean = 4.35, median = 3). These 102 extinct genera were distributed among the 22,760 genera and 163,022 species assessed by IUCN (in the version used here). Thus, only 0.45% of assessed genera are extinct, making these extinctions rare relative to overall (assessed) diversity, and even rarer relative to all 209,312 genera across life (0.05%).

These extinctions were strongly localized in their taxonomic distribution (Fig 1; S1 Table): the majority were in birds (*n* = 37) and mammals (*n* = 21). These represented 1.6% of recent bird genera and 1.6% of recent mammal genera. Genus-level extinctions were otherwise rare in vertebrates, including ray-finned fish (*n* = 4; only 0.08% of recent genera; less than one 10th of one percent), amphibians (*n* = 1; 0.18%), squamates (*n* = 2; 0.17%), and turtles (*n* = 1; 1.04%). Based on chi-squared tests, the differences in these frequencies were generally significant between birds and mammals and other vertebrate groups, except for turtles (S2 Table).

In these vertebrate groups, most genera in each group have one or more species that were assessed by IUCN (86%–100%; S1 Table). In other major groups with extinct genera, a much smaller percentage of genera have been assessed (arthropods = 3% of 117,978 genera; mollusks = 10% of 16,298 genera; plants = 32% of 21,466 genera). However, the percentages of assessed genera in those groups that have gone extinct are similar to those for amphibians, squamates, and fish. Specifically, 0.32% of assessed arthropod genera were extinct (*n* = 11 of 3,482 assessed genera) and 0.16% of assessed insect genera (*n* = 4 of 2,532), as were 0.77% of assessed mollusk genera (*n* = 13 of 1,698), and 0.17% of assessed plant genera (*n* = 12 of 6,939). Based on chi-squared tests (S2 Table), chordates and mollusks had significantly higher extinction frequencies than arthropods and plants, whereas mollusks and chordates were not significantly different from each other, nor were arthropods and plants.

Some groups with relatively few assessed genera had higher percentages (Ostracoda = 9.1% among 11 assessed genera; Bryophyta = 2.5%, *n* = 119; Arachnida = 1.7%, *n* = 350). However, among animal classes (*n* = 11), there was no significant relationship between the proportion of genera that were assessed by IUCN and the proportion of assessed genera that were extinct (*r*^2^ = 0.13, *P* = 0.27; ordinary and phylogenetic regression; S3 Table). Thus, the proportion of assessed genera within a clade does not appear to be a dominant factor that determines extinction levels and justifies including some clades and excluding others.

There have also been extinctions of entire families and orders (S1 Dataset). But family-level extinctions were confined to mammals (*n* = 5; 3% of families) and birds (*n* = 5; 2% of families), and order-level extinctions were confined to birds (*n* = 2; 5% of bird orders). Thus, the loss of higher taxa above the genus level is not a widespread pattern across life, or even across vertebrates.

Extinctions of genera (and other higher taxa) ultimately depend on species-level extinctions, but patterns in these extinctions need not be perfectly correlated. We obtained data on species-level extinctions from IUCN (989 species) and compared them to genus-level extinctions among major clades (S4 Table; S2 Dataset). Genus-level extinctions were most prevalent among birds (36% of extinct genera), mammals (21%), gastropods (12%), tracheophyte plants (9%), and arachnids (6%), whereas species-level extinctions were most common among gastropods (28% of extinct species), tracheophytes (17%), birds (17%), ray-finned fishes (10%), and mammals (9%). These extinction frequencies were significantly related among major groups (standard regression: *r*^2^ = 0.44; *P* = 0.0008; *n* = 22; phylogenetic regression: *r*^2^ = 0.42; *P* = 0.0019), but were clearly not identical. Furthermore, deleting groups with no or very few genus-level extinctions (1% or fewer of assessed genera) yields no significant relationship (standard and phylogenetic regression: *r*^2^ = 0.21; *P* = 0.2157; *n* = 9).

**Fig 2:**
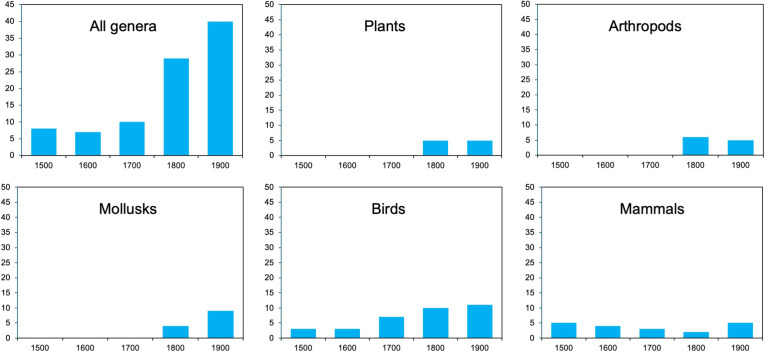
Patterns of genus-level extinctions over time among centuries. Each column shows the number of genus-level extinctions in that century. We show patterns only for those major groups with the most genus-level extinctions. Data for all groups are given in S2 Dataset (available on figshare: https://doi.org/10.6084/m9.figshare.27377613). We excluded the 2000s given that this century is not fully comparable to others (only 25% elapsed).

Over the centuries (1500s–1900s) and across all organisms (Fig 2; S3 Dataset), genus-level extinction rates increased over time, from low rates in the 1500s–1700s to a higher rate in the 1800s and the highest rate in the 1900s. Regressing century against extinctions per century for all organisms showed a significant, positive relationship (*r*^2^ = 0.83; *P* = 0.0303; *n* = 5; S3 Dataset). However, there was not a straightforward pattern of rapid acceleration across all groups. Arthropods had more extinctions in the 1800s than 1900s, and in plants, these two centuries were tied. Most genus-level extinctions were in mammals and birds. In mammals, the highest extinction rates were tied between the 1500s and 1900s. In birds, extinction rates were almost equal between the 1800s and 1900s.

**Fig 3:**
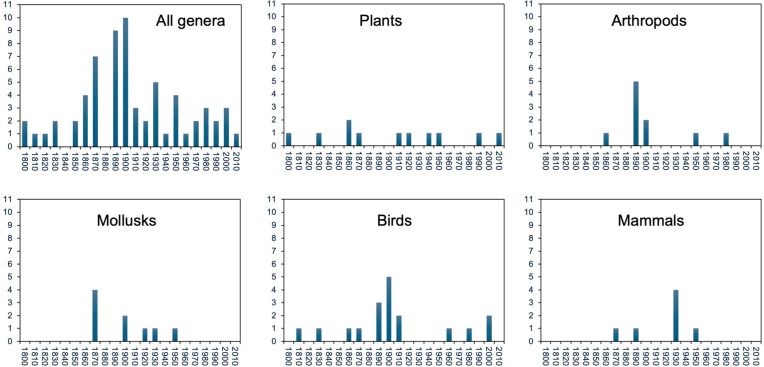
Patterns of genus-level extinctions over time among recent decades. Each column shows the number of genus-level extinctions in that decade. We show patterns only for those groups with the most genus-level extinctions, and only from the 1800s to 2010s. There were relatively few extinctions before the 1800s in most groups, except birds and mammals (Fig 2). Data for all groups are given in S3 Dataset (available on figshare: https://doi.org/10.6084/m9.figshare.27377613).

Rates at the decade-level (1800s–2000s) rejected a pattern of recent, accelerating genus-level extinction (Fig 3; S4 Dataset). Across all organisms, the three decades with the highest extinction rates were the 1900s, 1890s, and 1870s. Most extinctions were in birds and mammals. The highest rates in birds were in the 1900s, and in the 1930s in mammals. Plants, arthropods, and mollusks all showed maximum extinction rates in different decades of the late 1800s. Regressing rates against decades from the maximum in the 1890s and 1900s to the 2010s yielded a significant, negative relationship (*r*^2^ = 0.44; *P* = 0.0135; *n* = 13; S4 Dataset), showing that rates have recently decelerated toward the present rather than rapidly accelerating. Excluding the 2010s (with low extinction rates) yielded similar results (*r*^2^ = 0.40; *P* = 0.0271; *n* = 12).

**Fig 4:**
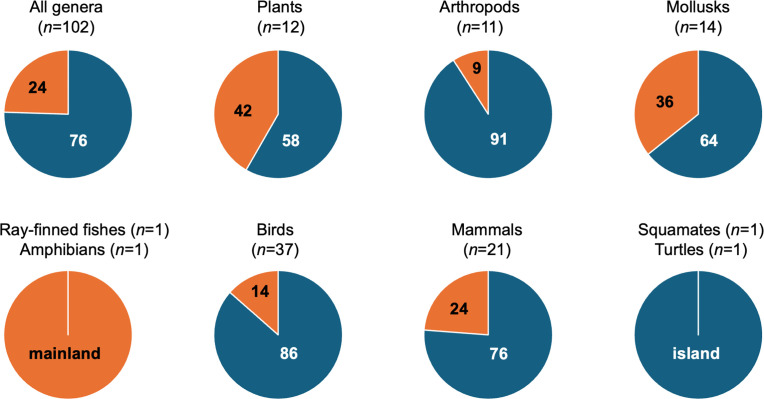
Distribution of genus-level extinctions on islands (blue) vs. the mainland (orange). Only genera that were entirely endemic to one or more islands are considered island taxa. The few marine genera were also counted as mainland. Results are only shown for selected groups. The full data are summarized in S2 Table. Data for each genus are given in S1 Dataset (available on figshare: https://doi.org/10.6084/m9.figshare.27377613).

Genus-level extinctions showed a straightforward spatial pattern (Fig 4; S5 Table; S1 Dataset): they were mostly in genera restricted to islands (76% of extinct genera across all groups). The majority of genus-level extinctions were of island endemics in arthropods (91%), insects (100%), mollusks (69%), and plants (58%). The most genus-level extinctions were in birds and mammals, and 86% of the genus-level bird extinctions were of island-endemic genera, as were 76% in mammals. All extinctions of families and orders were also of island endemics (S1 Dataset). All genus-level squamate and turtle extinctions were of island endemics.

There were exceptions to this pattern of island extinctions, but these were mostly in freshwater taxa (S1 Dataset). These included all four extinct genera of ray-finned fishes (0% on islands, 100% freshwater), the only extinct genus of amphibians (semi-freshwater), and the only non-island genus of extinct arthropods (a freshwater crustacean). Among the extinct, non-island mollusk genera (*n* = 4), all were freshwater. Further, among the five non-island genera of extinct birds, two were partially marine. Among the four extinct non-island mammal genera, one was marine and one was partially freshwater.

The overall pattern of island extinction differed significantly from expectations based on species richness patterns in plants and animals. It is estimated that ~20% of animal and plant species are island endemics, whereas ~80% are mainland [19,21]. Based on a chi-squared test, the observed frequencies of extinct genera across plants and animals were significantly different from this pattern (76% island versus 24% mainland, *n* = 102 genera: chi-squared = 61.638, *P* < 0.0001) as were the frequencies in animals (78% island versus 22% mainland, *n* = 90 genera: chi-squared = 57.829, *P* < 0.0001). The pattern in plants was not significant, presumably because of the smaller sample size and more similar frequencies (58% island versus 42% mainland, *n* = 12 genera; chi-squared = 61.638, *P* = 0.0917).

The island groups where most genus-level extinctions occurred depended on the higher taxon (S1 Dataset). For birds, most genus-level island extinctions were in the Mascarene Islands (31%; 10/32 extinct island endemic genera), Hawaiian Islands (28%; 9/32), and New Zealand (22%; 7/32). Most extinctions of mammal genera on island were in the West Indies (59%; 10/17). Extinctions of island mollusk genera were predominantly in the Mascarene Islands (38%; 3/8) and on Saint Helena (38%; 3/8). For arthropods, most were in the Seychelles (60%; 6/10). Plant extinctions were broadly distributed among island groups. Overall, these islands were mostly oceanic (e.g., Hawaiian, Mascarene) or were otherwise dominated by endemic taxa (e.g., Madagascar, New Zealand, Seychelles, West Indies).

We found 37 genera listed as “possibly extinct” (or “possibly extinct in the wild”) in the IUCN database (S5 Dataset). These genera were all monotypic except for one. There was also one possibly extinct family (Lipotidae, the Chinese river dolphin). Patterns among these genera were similar to those for extinct genera. Possibly extinct genera (S6 Table) were mostly distributed among vertebrates (*n* = 13), arthropods (*n* = 11), mollusks (*n* = 9), and plants (*n* = 4). In vertebrates, there were many in ray-finned fish (*n* = 7) but few in birds (*n* = 1) and mammals (*n* = 3). There was a general pattern of increasing rates among centuries (S1 Fig; S5 Dataset). Among decades (S2 Fig; S5 Dataset), the decade with the highest rate was the 1960s. The majority of possibly extinct genera occurred on islands (54%; S7 Table), especially in arthropods (82%). In vertebrates, many possibly extinct genera did not occur on islands, but these were mostly freshwater fishes. Similarly, in mollusks, all possibly extinct genera that were not island endemics occurred in freshwater.

Combining extinct and possibly extinct genera yielded results similar to those for extinct genera alone (S6 Dataset). The combined data yielded higher extinction levels (S8 Table), but the percentage of extinct genera among assessed genera remained below 0.5% for plants, insects, ray-finned fishes, amphibians, and reptiles, and was from 1% to 1.8% for mollusks, turtles, birds, and mammals. The fastest extinction rates were in the 1900s among centuries (S3 Fig). Among decades (S4 Fig), the fastest rates remained in the 1900s, 1890s, and 1870s, but with some relatively fast rates in some decades in the 1960s to 2000s. The fastest rates in animals, arthropods, tetrapods, and birds were in the 1890s and 1900s (decade). Extinctions were predominantly of island genera (70% overall; S9 Table), excepting mostly freshwater groups.

## Discussion

Human impacts over the last 500 years have led to the loss of hundreds of species of plants and animals [4–6,22]. The loss of higher taxa (e.g., genera, families, orders) is especially problematic, since these taxa may represent more evolutionary history than species alone (including monotypic higher taxa) and may be particularly distinct genetically, morphologically, and ecologically [7–12]. In an innovative paper, Ceballos and Ehrlich [17] examined recent extinctions of genera and other higher taxa, but only in tetrapod vertebrates (~2% of known species). They concluded that these extinctions were rapidly accelerating. They also suggested that these extinctions indicated a mass extinction event that could lead to the collapse of human civilization. Here, we have examined these extinctions of higher taxa more broadly, across living organisms.

Our conclusions are strikingly different. First, we found that genus-level extinctions were relatively rare. Like Ceballos and Ehrlich [17], we found many genus-level extinctions in birds and mammals but very few in non-avian reptiles and amphibians (Fig 1). But genus-level extinctions were also rare in every other major group (less than 1%), including ray-finned fishes (encompassing ~46% of vertebrate species), arthropods, mollusks, and plants (Fig 1). Hypothetically, the apparent rarity of genus-level extinctions in arthropods, mollusks, and plants might arise because these groups are not as thoroughly assessed as vertebrates. However, our extinction estimates account for this explicitly, by considering the proportion of extinctions only among genera assessed by IUCN. Furthermore, limited assessments do not explain why genus-level extinctions were rare across the majority of vertebrates (i.e., ray-finned fishes = 32,513 species, amphibians = 8,054, squamates = 11,769). Most genera in these groups have been assessed by IUCN (ray-finned fish = 86%, amphibians = 100%, squamates = 97%), as in birds and mammals (100% and 99%, respectively; S1 Table). There is also no significant relationship between extinction levels and the proportion of genera assessed within a group (S3 Table). A “mass extinction” should be widespread across life, not concentrated in two clades of modest size (birds = 10,677 species, mammals = 6,234 species). More importantly, even within birds and mammals, <2% of genera have actually gone extinct.

Second, within birds and mammals and other groups, genus-level extinctions were highly biased in their distribution (Fig 4). Specifically, we found that most (76%) genus-level extinctions were of island endemics. In birds and mammals, the frequency was even higher (birds = 86%, mammals = 79%). All extinctions of families (only in birds and mammals) and orders (only in birds) were also of island endemics. Many other groups showed this strong bias toward island extinctions (arthropods, mollusks, plants; Fig 4). Furthermore, most exceptions (e.g., ray-finned fishes, amphibians, crustaceans, some mollusks) were among freshwater and marine genera. Many authors have noted a widespread preponderance of species-level extinctions on islands [21–23]. Ceballos and Ehrlich [17] did not address this island bias, and instead stated that genus-level extinctions were concentrated in eastern North America and tropical regions of the Americas, Asia, and Africa. We suggest that these island extinctions should not be extrapolated to mainland taxa without careful justification. For example, invasive species are the primary cause of extinctions on islands, whereas overexploitation and habitat loss are as or more important on the mainland [21]. Perhaps most importantly, the extinction of a genus (most likely monotypic) on a single island or archipelago, while undeniably tragic, will presumably not strongly impact ecosystem services at the global scale. This island bias makes it seem unlikely that these genus-level extinctions are “destroying the conditions that make human life possible” [17].

Third, our results do not support the idea that genus-level extinctions are rapidly accelerating [17]. Those authors [17] only examined the cumulative number of genus-level extinctions over time, not extinctions in each time period (i.e., the extinction rate). They also only examined patterns among centuries. We found very different patterns when we examined extinction rates and when we considered the decadal timescale. At the century scale (Fig 2), we did find an overall increase in rates between the 1800s and earlier centuries and between the 1800s and 1900s when all groups were combined. But the increase between the 1800s and 1900s depended on the group, and was absent in some groups (e.g., arthropods, plants) and weak in others (e.g., birds). At the decadal scale (Fig 3), there was no evidence of accelerating rates toward the present within the last two centuries, and most groups showed their highest genus-level extinction rates in the late 1800s (plants, arthropods, mollusks) or early 1900s (tetrapods, birds, mammals). Thus, there was an overall pattern of decelerating extinction instead, specifically over the last ~100 years (Fig 3).

We found more potential evidence for increasing recent extinction rates among the 37 genera listed as “possibly extinct”, since their fastest overall extinction rates were in the 1960s. But combining the extinct and possibly extinct genera (S4 Fig) still showed the fastest extinction rates in the late 1800s and early 1900s (at the decadal timescale).

Ceballos and Ehrlich [17] suggested that extinction rates have increased relative to background rates from fossil mammal genera. Given recent anthropogenic extinctions of genera, recent extinction rates should be higher than rates before human impacts. However, the loss of genera (Fig 1) is <2% for birds and mammals, and <0.5% (among assessed genera) in most other large groups (ray-finned fishes, amphibians, squamates, arthropods, plants). This relatively low level of extinction seems inconsistent with a mass extinction event. Moreover, rates can be much faster when measured over shorter timescales [25–28], making it potentially problematic to compare rates measured over hundreds of years (i.e., recent anthropogenic rates) to those measured over millions of years (i.e., background rates in the fossil record). Thus, some researchers have argued for assessing mass extinctions based on absolute levels of species loss, and not by comparing extinction rates at very different timescales [29].

We acknowledge that there are important limitations to our study. First, there may be many extinct genera that were not included by the IUCN. This issue may be especially problematic in insects, in which relatively few genera have been assessed (2,532 out of 89,311; S1 Table), but which contain about half of all known species. Nevertheless, other groups also show low extinction levels for genera (<0.5%) despite being very large and being more thoroughly assessed by the IUCN (ray-finned fish, squamates, amphibians). Plants are not as thoroughly assessed as tetrapods, but they include many more species, and 32% of plant genera have been assessed. They show genus-level extinction frequencies (0.17%) broadly similar to those in insects (0.16%), mollusks (0.77%), and ray-finned fishes (0.08%). We also found no relationship between extinction levels and the proportion of genera assessed within a group (S3 Table). Furthermore, we found 32 insect genera in which the only species assessed by IUCN were extinct, despite there being other non-extinct species in these genera, and sometimes hundreds of them (S7 Dataset). Similarly, there are 10 insect families in which the only species assessed by IUCN are extinct. This pattern strongly suggests that IUCN is biased toward including extinct insect species, despite the relatively limited number of insect species assessed overall. Moreover, genera with extinct species in the IUCN database span most of the largest insect orders (e.g., Coleoptera, Diptera, Hemiptera, Lepidoptera, Orthoptera) and many smaller ones (Blattoidea, Dermaptera, Ephemeroptera, Phasmida, Plecoptera, Trichoptera). Thus, IUCN assessments were not confined to a few insect orders. On the other hand, this pattern does not guarantee that there are not additional extinct genera in insects and in other groups. Indeed, if IUCN sampling is unbiased regarding extinction (although this seems unlikely for insects), then one can extrapolate that approximately 139 additional insect genera have gone extinct (0.16% among 86,779 unassessed genera). We also acknowledge that there could be many dark extinctions [6,30–32], with many distinct clades going extinct before being described. However, it seems unlikely that past extinctions of taxa that were never known scientifically will lead to the future collapse of human civilization [17]. We also note that there can be considerable flux in the list of extinct species for a given group [22,33]. Yet, much of this flux comes from rediscovery of species previously considered extinct [22,33]. These rediscoveries would not overturn our main conclusions that genus-level extinctions are relatively rare, typically old, and largely confined to islands. We also showed that our overall conclusions were robust to including possibly extinct species. Therefore, they do not appear to be sensitive to the inclusion of additional extinct taxa. Similarly, we excluded Pleistocene extinctions and focused on the last 500 years, but large spikes in bird and mammal extinctions that occurred thousands of years ago (especially on islands [6]) would further reinforce our conclusion that these extinctions are biased toward birds and mammals and have decelerated recently.

Genus-level extinctions may also depend (at least partially) on how species are partitioned among genera. We assume that extinct genera were correctly treated as distinct and are not nested inside extant genera. Given this, they should (all else being equal) be more distinct phylogenetically than a randomly selected species within a genus, and possibly more distinct ecologically and morphologically. On the other hand, the human-based division of species among genera might impact the frequency and distribution of genus-level extinctions. For example, among the major groups of tetrapods (each with >95% of species assessed), genus-level extinctions were most prevalent among birds and mammals, followed by turtles. These are the three groups with the smallest species-per-genus ratios (4.6, 4.7, and 3.8, respectively) relative to amphibians (14.5) and squamates (10.2; ratios in S10 Table). Regressing the percentage of extinct genera against the species-per-genus ratios shows a strong negative relationship (*r*^2^ = 0.78; *P* = 0.0484–0.0487; *n* = 5; S11 Table), despite a small sample size. In groups with fewer species per genus, more genus-level extinctions are expected for the same number of species-level extinctions (all else being equal). Thus, this pattern may help explain why certain groups have more genus-level extinctions than others, and suggests that this may be related to arbitrary decisions about how species were assigned to genera (but note that this relationship becomes non-significant when including animal classes outside tetrapods; S11 Table). This arbitrary aspect also runs counter to the idea that these genus-level extinctions are part of a mass extinction event that could lead to the collapse of human civilization.

Other authors [34] have found that within birds and mammals (which contain the majority of genus-level extinctions), species extinctions occur disproportionately within smaller genera, and that extinctions of genera are more common than expected by chance alone from species extinctions. We are suggesting here that the prevalence of smaller genera in birds and mammals may help explain the high frequency of genus-level extinctions in these two groups relative to other tetrapods. Our results are also potentially consistent with the idea that there are more extinctions of species in small genera because small genera may tend to occur on islands [34].

There may be many additional genus-level extinctions in the future, and our study should not be interpreted as downplaying current threats to biodiversity. However, it is unclear if the past extinctions described here are relevant to predicting these future extinctions. After all, most of these past genus-level extinctions were on islands and occurred many decades ago. For example, climate change may be a major cause of future extinctions [35–38] but is not a major cause of recent past extinctions or current IUCN-listed threats among species [39]. Similarly, invasive species were the primary cause of the majority of species extinctions on islands [21], but habitat loss is the greatest current threat to presently endangered species, both on islands and on the mainland [21].

Some readers may be surprised that genus-level extinction rates appear to have declined over the last ~100 years (Fig 3). Several factors may contribute to this pattern. First, most genus-level extinctions were of birds and mammals, and conservation action has demonstrably slowed recent extinctions in these two groups [40]. Second, many of the most vulnerable genera may have already gone extinct, leaving behind a pool of genera that are generally more resilient (at least to the most prevalent threats in the recent past, like invasive species on islands). Third, an apparent slowdown might be an artifact of delays in adding newly extinct genera to the IUCN list. However, the apparent slowdown in genus-level extinction rates spans >100 years (Fig 3), and new extinct genera were still added in the 2000s and 2010s. Therefore, this latter factor seems unlikely to be the sole explanation.

In summary, our study shows that there has been recent extinctions of genera in many different groups of animals and plants. However, these extinctions were relatively rare: they occurred in less than 2% of bird and mammal genera, about 1% in turtles, and less than 0.5% of assessed genera in most other groups. Most extinct genera were monotypic, and there were no extinctions of families outside birds and mammals and no extinctions of orders outside birds. Furthermore, rather than showing rapid acceleration toward the present, these genus-level extinctions have generally decelerated after reaching their highest rate ~100 years ago or more. We also find that these extinctions were mostly of island endemics. Based on these results, we do not think that these genus-level extinctions are “destroying the conditions that make human life possible” and will presumably not cause the “collapse of civilization” [17]. Indeed, Ceballos and Ehrlich [17] provided no evidence linking their results on genus-level extinctions to global ecosystem services or the collapse of civilization. We think that the reason why future extinctions must be halted is not only because of their potential consequences for humans (which might be trivial for many narrowly distributed island endemics), but because all living things have inherent value and driving other species and genera to extinction is morally wrong [41,42]. Therefore, every human-related extinction should be prevented, regardless of whether these extinctions are part of a mass extinction and regardless of whether every species is directly beneficial to humans. Moreover, given current skepticism toward conservation and science in general, we think it is especially important to be rigorous and accurate about current threats to biodiversity and their consequences for humanity, without overstatement or exaggeration.

## Methods

Following previous studies on recent extinctions [4,17,29], we utilized the IUCN database [18]. The version used included assessments for 163,022 species and 22,760 genera. The IUCN provides assessments across all major groups of macroscopic organisms (plants, animals, fungi), even though it does not include all species within every group. IUCN aims to use standardized criteria for categorizing species as extinct and threatened across groups [43]. However, there is still variability in the application of these criteria in individual cases. For example, among species listed as extinct, search efforts were mentioned for ~45% of the species and the length of time each species was missing was mentioned in ~50% [44]. We acknowledge that there are other lists of extinct species besides the IUCN for specific groups (e.g., [22,33,45]), but these do not offer comparable assessments across groups similar to IUCN. We used the IUCN list of “possibly extinct” genera as a way to assess the robustness of our conclusions to the addition of more extinct genera.

We downloaded the final IUCN database used on 27 June 2024. We then searched these data for species listed as “extinct” or “extinct in the wild” (which were both treated as “extinct”). We next sorted the IUCN database to find those genera in which all IUCN-assessed species were categorized as extinct.

We then used the Catalogue of Life (CoL; [19]) to determine the total number of described species in each genus. Genera in which the number of extinct species matched the number of described species were considered extinct. These were generally monotypic genera. For many other genera (especially in insects), the only species in the genus that was assessed by IUCN was extinct and there were one or more extant species. These genera were not considered extinct. We list these excluded taxa in S7 Dataset.

Some genera with one or more extinct species were not listed on the CoL. We found all these genera listed instead on Global Biodiversity Information Facility (GBIF; gbif.org). For tetrapods, we also checked our list of extinct genera against that of Ceballos and Ehrlich [17]. Thus, we identified several extinct genera that were not on our list from IUCN. These were added. We also identified genera listed as extinct by Ceballos and Ehrlich [17] that had one or more extant species in the CoL. These were not included. All these discrepancies are listed and explained in S8 Dataset.

To infer the percentage of extinct genera for each major group, we estimated the number of extinct genera relative to the overall number of genera in the group that were assessed by IUCN. The number of genera assessed by IUCN in each major group is given in S9 Dataset. We listed a genus as assessed by IUCN if one or more species were assessed, but in some genera only some species were assessed. For example, for many insect genera, the only species listed by IUCN is extinct, even though the genus contains many living species.

We compared extinction frequencies among taxonomic groups. We generally followed the classification used by the IUCN and CoL. Thus, we considered “plants” to correspond to the Kingdom Plantae, corresponding to the Archaeplastida (and thus including Rhodophyta, along with Bryophyta and Tracheophyta, among the plant taxa included here). We performed chi-squared tests (in R version 4.3.1 [46]) to assess whether frequencies were significantly different between groups. We primarily focused on animal classes. We recognize that classes are arbitrary ranks, but they correspond to the major groups that are often treated separately in similar analyses (e.g., amphibians, birds, mammals, insects). Squamates, turtles, and birds were treated as separate classes. We also compared animal phyla to plants. Plants with extinct genera primarily belonged to two major groups (Bryophyta and Tracheophyta).

We tested whether the proportion of assessed genera within a taxonomic class was significantly related to the proportion of extinct genera within a class (to determine whether results from less well-assessed clades were strongly biased by this factor). We performed ordinary least squares (OLS) regression (in R) among animal classes with one or more extinct genera (*n* = 11). The proportion of extinct genera was the dependent variable and the proportion of assessed genera was the independent variable.

A phylogenetic correction was potentially problematic, given that recent, human-related factors (IUCN assessments, recent extinctions, genus-level taxonomy) cannot be phylogenetically inherited among species or clades. Nevertheless, we confirmed that we obtained similar results using phylogenetic generalized least-squares (PGLS) regression [47], implemented in the R package *caper* [48]. We used three time-calibrated animal phylogenies (from [49]), pruned to include a single representative tip from each class. Given a time-calibrated tree with one tip per clade, the choice of tips does not impact the resulting branch lengths. Trees were pruned using the R package *ape* [50]. These trees did not include Ostracoda, but we used a tip from Malacostraca instead. These two classes do not have identical phylogenetic positions when all arthropod classes are included [51], but given that only three arthropod classes were included here, both are closer to insects than arachnids (yielding identical phylogenetic positions).

We also compared patterns in genus-level extinctions and species-level extinctions among major groups. Extinctions of higher taxa depend on species-level extinctions, but it is unclear how closely patterns of species-level and genus-level extinctions are related. We obtained the list of extinct species from IUCN [18]. We used the list of extinct species “as is”, and only excluded the three genera listed below (but we acknowledge that additional species may be rediscovered or invalid). We summarized extinct species and extinct genera among animal classes and plant phyla. We estimated the proportion of all species-level extinctions that occurred in each group, and the proportion of all genus-level extinctions. We then tested for a relationship between these proportions among groups using regression. We used both OLS regression and PGLS. Again, we generally used taxonomic classes as units of analyses. However, for some animal phyla, there was only a single extinct species (Annelida, Nemertea, Platyhelminthes), and so these were used instead (using classes would yield identical results). For plants, we also used phyla (Bryophyta, Rhodophyta, Tracheophyta): for genus-level extinctions, use of classes would involve the same units.

For this PGLS analysis, we used the Timetree of Life compilation (timetree.org) to obtain a time-calibrated phylogeny among these 22 groups, spanning plants and animals. For many groups, there were extinct species but no extinct genera, or genus-level frequencies were very low (1% or less). Therefore, we also performed an analysis excluding these groups (*n* = 9 included).

All phylogenies used are given in S10 Dataset. All statistical analyses were performed in R [46] version 4.3.1. Samples of code used are given in S11 Dataset.

We examined patterns of extinction over time. To infer the approximate timing of extinction for each genus, we used information from IUCN on when each species was last recorded. Given the potential uncertainty about the exact timing of extinction based on dates of last appearance, dates were only assigned to decades and centuries (rather than with greater precision). When dates were given as multiple decades, the decade was considered unknown. When dates were given as multiple centuries, both decade and century were considered unknown. For extinct higher taxa containing multiple species, we used the latest date to represent its extinction (i.e., a genus does not go extinct until the last species has gone extinct). We note that for most genera, the year last seen and the year last recorded were identical. In cases in which these differed, we generally used the year last recorded. The only exceptions were three genera (birds: *Fregilupus, Mundia*; mammal: *Hydrodamalis*) in which the last year recorded differed from the inferred timing of extinction, and we used the latter.

Some species were listed by IUCN but lacked a value for when the species was last recorded. For these species, we conducted the following steps until this information was found. First, for the few extinct tetrapod species not listed by IUCN, we used the inferred extinction dates from Ceballos and Ehrlich [17]. For other species, we first reviewed the entire IUCN listing, which often included a year last seen. We next reviewed the citations in the IUCN listing. Then, for species in the Americas, we checked NatureServe Explorer (https://explorer.natureserve.org/). Next, we consulted bibliographies of species accounts in Wikipedia (http://wikipedia.org/) and a website about Anthropocene extinctions (http://deadasthedodo.com/) to find relevant articles. Finally, we searched Google Scholar using the keywords “extinction” and the species name. All these searches were conducted in May of 2024 (based on an earlier version of the IUCN list, with an identical set of extinct species). Overall, we were able to assign an approximate extinction date for all but 5 genera (S1 Dataset).

Through this process, we also found that two genera listed as extinct by IUCN have been rediscovered (the insect *Phyllococcus* [52] and the snail *Cyclosurus* [33]). We also found that the extinct mollusk genus *Collisella* is part of the extant genus *Lottia* [53]. All three genera were excluded, and the overall number of assessed gastropod, mollusk, and animal genera were each reduced by one because of *Collisella*.

We summarized the number of extinctions (based on the date last recorded) in each century and in each decade. From this summary, we could infer which centuries and decades had the highest extinction rates (extinctions per time period), and whether rates were generally increasing or decreasing over time. We did this across living organisms and within major groups. For centuries, we only focused on extinctions from the 1500s through the 1900s. There were no extinct higher taxa in our dataset from before 1500. Given that we are only partway through the 2000s, numbers from this century are not directly comparable to the others. For decades, we looked at the 1800s to the 2010s. There were relatively few extinctions before the 1800s. Extinction rates in the 2010s might be underestimated due to relatively short time periods between the last sighting of a species and when it is considered extinct.

Ceballos and Ehrlich [17] gave an exact year for the extinction of many species and genera, but they did not explain how these dates were obtained. However, their dates appeared to match the “year last recorded” given by IUCN. Thus, our data on the timing of extinction should be comparable to theirs.

We did not “correct” these analyses of extinctions over time based on the year in which the genus (or other higher taxa) was described. Hypothetically, more extinctions might be expected in more recent centuries and decades, under the assumption that taxa can only go extinct after they have been formally described. Yet, we found that this assumption was not supported. To address this, we determined when each genus was first described, based on the year the oldest species in the genus was described (even if it was initially described as belonging to a different genus). We obtained description dates from the CoL (for most genera) and from GBIF. These dates are reported in S1 Dataset. We then compared these dates to the decade last seen. Contrary to the assumption that extinction dates must come after description dates, we found that many genera became extinct before they were scientifically described (*n* = 33; mean ~165 years before), almost as many as became extinct after they were described (*n* = 38; mean ~154 years after). In other cases, the dates last seen were were unclear (*n* = 17) or very close to the description date (same decade; *n* = 14).

We tested whether rates significantly increased over the centuries and decreased in recent decades. We regressed the extinction rate per century against the century (excluding the 2000s) using the data for all groups combined. We also tested whether there was a significant deceleration in extinction rates from the peak extinction rates (1890s–1900s) toward the present (2010s). We regressed the decade against the inferred extinction rate of that decade, using the combined data across all groups. This test addressed whether the apparent decline in rates over this period was distinguishable from stochastic variation. The expectation from Ceballos and Ehrlich [17] was that rates should significantly increase over this time period (not decline), given their conclusion that extinction rates are rapidly accelerating.

We then determined whether each genus was an island endemic or not (S1 Dataset). This was determined primarily through the verbal descriptions of species distributions in the species accounts from IUCN. We found only one extinct genus in which some species were island endemics and others were not. Since the genus overall was not an island endemic, we treated the genus as mainland. The few marine genera were also treated as non-island. We considered all islands (i.e., land surrounded by water) that were named as islands to be islands: we did not attempt to bias or predetermine our results by only considering certain types of islands to be islands (e.g., oceanic versus continental). Nevertheless, we did document the island group where each extinct genus occurred (S1 Dataset). We found (see Results) that most of the relevant island groups where generic extinctions occurred were oceanic (Hawaiian, Mascarene) or continental islands with high levels of endemism (Madagascar, New Zealand, Seychelles, West Indies).

We tested whether the frequency of extinctions on islands differed significantly from the pattern expected based on the relative species richness of islands and continental regions, using chi-squared tests. The relative frequency of mainland versus island species is estimated to be roughly 80% mainland versus 20%, for both plants and animals [21,23]. We did not know the frequencies for genera specifically, but we assumed that these would be broadly similar to those for species. We performed the two-sample test for equality of proportions with a continuity correction.

We also examined the IUCN habitat classification (“system”), which characterizes species as terrestrial, freshwater, or marine (or a combination of two or more habitats). Data for each genus are given in S1 Dataset.

We then examined patterns among “possibly extinct” genera (S5 Dataset). These typically consisted of one or more species that have not been seen for decades, but lacked sufficient evidence to be categorized as extinct. We used the IUCN classification of species as “critically endangered, possibly extinct” and “critically endangered, possibly extinct in the wild.” Finally, we examined the results after combining the extinct and possibly extinct species, in terms of extinctions among clades, extinctions over time, and extinctions on islands (S6 Dataset).

In the Discussion, we addressed whether the proportion of extinct genera within a class (among all assessed genera) was related to the ratio of species to genera within a class. We performed OLS and PGLS regression primarily within tetrapods (*n* = 5), to compare birds and mammals to closely related groups. We also analyzed vertebrates (*n* = 6) and all animals (*n* = 11). We used the pruned trees described above, with new trees pruned to include only tetrapod and vertebrates (note that the three trees are identical within vertebrates). All trees used are available in S11 Dataset.

## Supporting information

S1 Table Patterns of recent, genus-level extinctions among major groups of animals and plants. (DOCX)

S2 Table Statistical comparisons of extinction frequencies among major groups. (DOCX)

S3 Table Relationships between the proportions of assessed genera and extinct genera among animal classes. (DOCX)

S4 Table Patterns in species-level extinctions, compared to genus-level extinctions. (DOCX)

S5 Table The proportion of extinct genera in each taxonomic group that are island endemics. (DOCX)

S6 Table Distribution of possibly extinct genera among major groups of animals and plants. (DOCX)

S7 Table The proportion of possibly extinct genera in each taxonomic group that are island endemics. (DOCX)

S8 Table Distribution of extinct and possibly extinct genera among major groups of animals and plants. (DOCX)

S9 Table The proportion of extinct and possibly extinct (EPE) genera in each taxonomic group that are island endemics. (DOCX)

S10 Table The ratio of species to genera among major groups of animals and plants. (DOCX)

S11 Table Relationships between extinction frequencies and genus-to-species ratios. (DOCX)

S1 Fig Patterns of genus-level extinctions over time among centuries for possibly extinct genera. (DOCX)

S2 Fig Patterns of genus-level extinctions over time among decades for possibly extinct genera. (DOCX)

S3 Fig Patterns of genus-level extinctions over time among centuries for the combined sets of extinct and possibly extinct genera. (DOCX)

S4 Fig Patterns of genus-level extinctions over time among decades for the combined sets of extinct and “possibly extinct” genera. (DOCX)

S1 Dataset Data on extinct genera, families, and orders. (XLSX)

S2 Dataset Data on extinct species. (XLSX)

S3 Dataset The number of genera inferred to have gone extinct in each century in each group. (XLSX)

S4 Dataset The number of genera inferred to have gone extinct in each decade in each group. (XLSX)

S5 Dataset Data on possibly extinct genera and families. (XLSX)

S6 Dataset Combined data on extinct and possibly extinct genera over time. (XLSX)

S7 Dataset Excluded genera and families. (XLSX)

S8 Dataset Resolving discrepancies between datasets. (XLSX)

S9 Dataset The number of genera assessed by IUCN. (XLSX)

S10 Dataset R code used. (TXT)

S11 Dataset Phylogenetic trees used. (NEXUS)

## Abbreviations

**:**
GBIF: Global Biodiversity Information Facility
IUCN: International Union for the Conservation of Nature
OLS: ordinary least squares regression;
PGLS: phylogenetic generalized least-squares regression.
