# Supplementary material for: Recent extinctions of plant and animal genera are rare, localized, and decelerated
Source: PLoS Biol. 2025 Sep 4;23(9):e3003356. doi: 10.1371/journal.pbio.3003356 (PMC12410804; doi:10.1371/journal.pbio.3003356)
Supplement: S1 Table — (DOCX) [file pbio.3003356.s001.docx]

**S1 Table.** **Patterns of recent, genus-level extinctions among major groups of animals and plants.** For each group, we give the number of recently extinct genera in that group (Ext. genera), the total number of genera with one or more species assessed by IUCN (Assd.), the proportion of extinct genera (among those assessed; Ext./Assd.), the proportion of genera assessed in the group relative to the total number of described genera (Assd./Total) in the group, the total number of species in the group (Total species), and the proportion of extinct genera among all genera in the group (Extinct/Total). Note that we reduced the number of assessed genera to account for the invalid gastropod genus *Collisella* in IUCN, but did not modify the number of total genera or species (since this genus is not recognized by the Catalogue of Life).

| Taxon | Ext. genera | Assd. | Ext./  Assd. | Assd./Total | Total genera | Total species | Extinct/Total |
| --- | --- | --- | --- | --- | --- | --- | --- |
| All | 102 | 22,760 | 0.0045 | 0.1087 | 209,312 | 2,170,160 | 0.00049 |
| Animalia | 90 | 15,478 | 0.0058 | 0.0940 | 164,622 | 1,553,708 | 0.00055 |
| Arthropoda | 11 | 3482 | 0.0032 | 0.0295 | 117,978 | 1,204,321 | 0.00009 |
| Arachnida | 6 | 350 | 0.0171 | 0.0364 | 9609 | 95,970 | 0.00062 |
| Ostracoda | 1 | 11 | 0.0909 | 0.0030 | 3645 | 17,050 | 0.00027 |
| Insecta | 4 | 2532 | 0.0016 | 0.0284 | 89,311 | 995,088 | 0.00004 |
| Chordata | 66 | 9998 | 0.0066 | 0.9254 | 10,804 | 74,220 | 0.00611 |
| Actinopterygia | 4 | 4276 | 0.0009 | 0.8586 | 4980 | 32,513 | 0.00080 |
| Amphibians | 1 | 558 | 0.0018 | 1.0072 | 554 | 8,054 | 0.00180 |
| Birds | 37 | 2396 | 0.0154 | 1.0381 | 2308 | 10,677 | 0.01603 |
| Mammals | 21 | 1308 | 0.0161 | 0.9864 | 1326 | 6234 | 0.01584 |
| Squamates | 2 | 1122 | 0.0018 | 0.9723 | 1154 | 11,769 | 0.00173 |
| Turtles | 1 | 92 | 0.0109 | 0.9583 | 96 | 365 | 0.01042 |
| Mollusks | 13 | 1698 | 0.0077 | 0.1042 | 16,294 | 138,354 | 0.00080 |
| Bivalves | 1 | 183 | 0.0055 | 0.0562 | 3255 | 23,883 | 0.00031 |
| Gastropods | 12 | 1345 | 0.0089 | 0.1378 | 9762 | 100,228 | 0.00123 |
| Plantae | 12 | 6939 | 0.0017 | 0.3233 | 21,466 | 385,797 | 0.00056 |
| Bryophyta | 3 | 119 | 0.0252 | 0.1137 | 1047 | 12,243 | 0.00286 |
| Tracheophyta | 9 | 6702 | 0.0013 | 0.4015 | 16,694 | 365,207 | 0.00054 |
