# Supplementary material for: Recent extinctions of plant and animal genera are rare, localized, and decelerated
Source: PLoS Biol. 2025 Sep 4;23(9):e3003356. doi: 10.1371/journal.pbio.3003356 (PMC12410804; doi:10.1371/journal.pbio.3003356)
Supplement: S2 Table — (DOCX) [file pbio.3003356.s002.docx]

**S2 Table.** Statistical comparisons of extinction frequencies among major groups. We performed chi-squared tests in R. We compared major groups of chordates and different animal phyla and plants. We did not compare groups with nearly identical frequencies of extinction (e.g. mammals vs. birds).

| Group 1 | Group 2 | chi-squared | P-value |
| --- | --- | --- | --- |
| Within chordates |  |  |  |
| Mammals (1.6%) | Actinopterygians (0.09%) | 48.037 | 4.183e-12 |
| Birds (1.5%) | Actinopterygians (0.09%) | 50.565 | 1.153e-12 |
| Mammals (1.6%) | Squamates (0.18%) | 11.644 | 0.0006 |
| Birds (1.5%) | Squamates (0.18%) | 11.790 | 0.0006 |
| Mammals (1.6%) | Amphibians (0.18%) | 5.660 | 0.0174 |
| Birds (1.5%) | Amphibians (0.18%) | 5.610 | 0.0179 |
| Mammals (1.6%) | Turtles (1.1%) | <0.0001 | 1.0000 |
| Birds (1.5%) | Turtles (1.1%) | <0.0001 | 1.0000 |
| Squamates (0.18%) | Turtles (1.1%) | 0.355 | 0.5515 |
|  |  |  |  |
| Animal phyla and plants |  |  |  |
| Chordates (0.67%) | Arthropods (0.32%) | 4.799 | 0.0285 |
| Chordates (0.67%) | Plants (0.17%) | 20.160 | 7.123e-06 |
| Chordates (0.67%) | Mollusks (0.78%) | 0.109 | 0.7411 |
| Plants (0.17%) | Arthropods (0.32%) | 1.552 | 0.2129 |
| Mollusks (0.78%) | Arthropods (0.32%) | 4.078 | 0.0435 |
| Mollusks (0.78%) | Plants (0.17%) | 14.613 | 0.0001 |
