# Supplementary material for: Recent extinctions of plant and animal genera are rare, localized, and decelerated
Source: PLoS Biol. 2025 Sep 4;23(9):e3003356. doi: 10.1371/journal.pbio.3003356 (PMC12410804; doi:10.1371/journal.pbio.3003356)
Supplement: S3 Table — (DOCX) [file pbio.3003356.s003.docx]

**S3 Table.** Relationships between the proportions of assessed genera and extinct genera among animal classes. We tested relationships among the 11 classes with one or more extinct genera. We used both ordinary least-squares regression (OLS) and phylogenetic generalized least squares regression (PGLS). For PGLS, we used three phylogenies (Trees 1–3) with different branch lengths. Note that for Tree 1, the maximum likelihood estimation of lambda failed. For Trees 2 and 3, the estimated lambda was 0, and therefore Tree 1 we used a comparable value (0.01).

| Method | r^2^ | P | Estimate |
| --- | --- | --- | --- |
| OLS | 0.13 | 0.2694 | -0.0195 |
| PGLS |  |  |  |
| Tree 1 | 0.13 | 0.2728 | -0.0195 |
| Tree 2 | 0.13 | 0.2694 | -0.0195 |
| Tree 3 | 0.13 | 0.2694 | -0.0195 |
