# Supplementary material for: Recent extinctions of plant and animal genera are rare, localized, and decelerated
Source: PLoS Biol. 2025 Sep 4;23(9):e3003356. doi: 10.1371/journal.pbio.3003356 (PMC12410804; doi:10.1371/journal.pbio.3003356)
Supplement: S4 Table — (DOCX) [file pbio.3003356.s004.docx]

**S4 Table.** Patterns in species-level extinctions, compared to genus-level extinctions. We show the number of extinct species from IUCN in each major group (Dataset S2), the proportion of all extinct species belonging to each major group, the proportion of extinct genera (from Dataset S1), the total number of species in each group (), and the number of species in each group that were assessed by IUCN.

| Taxon | Extinct species | Proportion extinct species | Proportion extinct genera | Total Species | Assessed  species |
| --- | --- | --- | --- | --- | --- |
| Animalia |  |  |  |  |  |
| Annelida | 2 | 0.0020 | 0 | 17766 | 359 |
| Arthropoda |  |  |  |  |  |
| Arachnida | 9 | 0.0091 | 0.0588 | 95,970 | 774 |
| Diplopoda | 3 | 0.0030 | 0 | 17,050 | 250 |
| Hexanauplia | 1 | 0.0010 | 0 | NA | NA |
| Insecta | 58 | 0.0588 | 0.0392 | 995,088 | 12,718 |
| Malacostraca | 8 | 0.0081 | 0 | 45953 | 3041 |
| Maxillopoda | 1 | 0.0010 | 0 | NA | NA |
| Ostracoda | 2 | 0.0020 | 0.0098 | 17,050 | 14 |
| Chordata |  |  |  |  |  |
| Actinopterygia | 94 | 0.0953 | 0.0392 | 32,513 | 26599 |
| Amphibia | 39 | 0.0396 | 0.0098 | 8,054 | 8011 |
| Aves | 164 | 0.1663 | 0.3627 | 10,677 | 11195 |
| Chondrichthyes | 1 | 0.0010 | 0 | 1282 | 1247 |
| Mammalia | 85 | 0.0862 | 0.2059 | 6234 | 5983 |
| Squamata | 26 | 0.0264 | 0.0196 | 11,769 | 1013 |
| Testudines | 8 | 0.0081 | 0.0098 | 365 | 272 |
| Mollusca |  |  |  |  |  |
| Bivalvia | 32 | 0.0325 | 0.0098 | 23,883 | 833 |
| Gastropoda | 275 | 0.2789 | 0.1176 | 100,228 | 7516 |
| Nemertea | 1 | 0.0010 | 0 | 1385 | 6 |
| Platyhelminthes | 1 | 0.0010 | 0 | 26,424 | 2 |
| Plantae |  |  |  |  |  |
| Bryophyta | 4 | 0.0041 | 0.0294 | 12,243 | 210 |
| Rhodophyta | 1 | 0.0010 | 0 | 7531 | 78 |
| Tracheophyta | 171 | 0.1734 | 0.0882 | 365,207 | 70584 |
