# Supplementary material for: Recent extinctions of plant and animal genera are rare, localized, and decelerated
Source: PLoS Biol. 2025 Sep 4;23(9):e3003356. doi: 10.1371/journal.pbio.3003356 (PMC12410804; doi:10.1371/journal.pbio.3003356)
Supplement: S5 Table — (DOCX) [file pbio.3003356.s005.docx]

**S5 Table.** The proportion of extinct genera in each taxonomic group that are island endemics. Full data are in Dataset S1. One extinct genus that had one island species and one mainland species was treated as mainland (i.e. the genus was not an island endemic).

| Taxon | Extinct genera | Island | Mainland | Proportion island |
| --- | --- | --- | --- | --- |
| All | 102 | 77 | 25 | 0.755 |
| Animalia | 90 | 70 | 20 | 0.778 |
| Arthropoda | 11 | 10 | 1 | 0.909 |
| non-insect arthropods | 7 | 6 | 1 | 0.857 |
| Arachnida | 6 | 6 | 0 | 1.000 |
| Ostracoda | 1 | 0 | 1 | 0.000 |
| Insecta | 4 | 4 | 0 | 1.000 |
| Chordata | 66 | 51 | 15 | 0.773 |
| Actinopterygians | 4 | 0 | 4 | 0.000 |
| Amphibians | 1 | 0 | 1 | 0.000 |
| Birds | 37 | 32 | 5 | 0.865 |
| Mammals | 21 | 16 | 5 | 0.762 |
| Squamates | 2 | 2 | 0 | 1.000 |
| Turtles | 1 | 1 | 0 | 1.000 |
| Mollusks | 13 | 9 | 4 | 0.692 |
| Bivalves | 1 | 1 | 0 | 1.000 |
| Gastropods | 12 | 8 | 4 | 0.667 |
| Plantae | 12 | 7 | 5 | 0.583 |
| Bryophyta | 3 | 1 | 2 | 0.333 |
| Tracheophyta | 9 | 6 | 3 | 0.667 |
