# Supplementary material for: Recent extinctions of plant and animal genera are rare, localized, and decelerated
Source: PLoS Biol. 2025 Sep 4;23(9):e3003356. doi: 10.1371/journal.pbio.3003356 (PMC12410804; doi:10.1371/journal.pbio.3003356)
Supplement: S6 Table — (DOCX) [file pbio.3003356.s006.docx]

**S6 Table.** Distribution of possibly extinct genera among major groups of animals and plants. For each group, we give the number of possibly extinct genera in that group (PE genera), the total number of genera assessed by IUCN (Assd.), the proportion of possibly extinct genera (among those assessed; PE/Assd.), the number of genera assessed in the group relative to the total number of genera in the group, the total number of species in the group, and the proportion of possibly extinct genera among all genera in the group.

| Taxon | PE genera | Assd. | PE/  Assd. | Assd./Total | Total genera | Total species | PE/Total |
| --- | --- | --- | --- | --- | --- | --- | --- |
| All | 37 | 22,760 | 0.0016 | 0.1087 | 209,312 | 2170160 | 0.00018 |
| Animalia | 33 | 15,478 | 0.0021 | 0.0940 | 164,622 | 1,553,708 | 0.00020 |
| Arthropoda | 11 | 3482 | 0.0032 | 0.0295 | 117,978 | 1,204,321 | 0.00009 |
| Arachnida | 3 | 350 | 0.0086 | 0.0364 | 9609 | 95,970 | 0.00031 |
| Diploda | 1 | 48 | 0.0208 | 0.0132 | 3645 | 17,050 | 0.00027 |
| Insecta | 7 | 2532 | 0.0028 | 0.0284 | 89,311 | 995,088 | 0.00008 |
| Chordata | 13 | 9998 | 0.0013 | 0.9254 | 10,804 | 74,220 | 0.00120 |
| Actinopterygia | 7 | 4276 | 0.0016 | 0.8586 | 4980 | 32,513 | 0.00141 |
| Amphibians | 1 | 558 | 0.0018 | 1.0072 | 554 | 8,054 | 0.00181 |
| Birds | 1 | 2396 | 0.0004 | 1.0381 | 2308 | 10,677 | 0.00043 |
| Mammals | 3 | 1308 | 0.0023 | 0.9864 | 1326 | 6234 | 0.00226 |
| Squamates | 1 | 1122 | 0.0009 | 0.9723 | 1154 | 11,769 | 0.00087 |
| Mollusks | 9 | 1698 | 0.0053 | 0.1042 | 16,294 | 138,354 | 0.00055 |
| Bivalves | 1 | 183 | 0.0055 | 0.0562 | 3255 | 23,883 | 0.00031 |
| Gastropods | 8 | 1345 | 0.0060 | 0.1378 | 9762 | 100,229 | 0.00082 |
| Plantae | 4 | 6939 | 0.0006 | 0.3233 | 21,466 | 385,797 | 0.00019 |
| Rhodophyta | 1 | 46 | 0.0217 | 0.0372 | 1235 | 7,531 | 0.00081 |
| Tracheophyta | 3 | 6702 | 0.0004 | 0.4015 | 16,694 | 365,207 | 0.00018 |
