# Supplementary material for: Recent extinctions of plant and animal genera are rare, localized, and decelerated
Source: PLoS Biol. 2025 Sep 4;23(9):e3003356. doi: 10.1371/journal.pbio.3003356 (PMC12410804; doi:10.1371/journal.pbio.3003356)
Supplement: S7 Table — (DOCX) [file pbio.3003356.s007.docx]

**S7 Table.** The proportion of possibly extinct genera in each taxonomic group that are island endemics. Full data are in Dataset S4.

| Taxon | Extinct genera | Island | Mainland | Proportion island |
| --- | --- | --- | --- | --- |
| All | 37 | 20 | 17 | 0.541 |
| Animalia | 33 | 20 | 16 | 0.545 |
| Arthropoda | 11 | 9 | 2 | 0.818 |
| Arachnida | 3 | 3 | 0 | 1.000 |
| Diplopoda | 1 | 1 | 0 | 1.000 |
| Insecta | 7 | 5 | 2 | 0.714 |
| Chordata | 13 | 4 | 9 | 0.308 |
| Actinopterygians | 7 | 2 | 5 | 0.286 |
| Amphibians | 1 | 0 | 1 | 0.000 |
| Birds | 1 | 1 | 0 | 1.000 |
| Mammals | 3 | 0 | 3 | 0.000 |
| Squamates | 1 | 1 | 0 | 1.000 |
| Mollusks | 9 | 5 | 4 | 0.556 |
| Bivalves | 1 | 0 | 1 | 0.000 |
| Gastropods | 8 | 5 | 3 | 0.625 |
| Plantae | 4 | 3 | 1 | 0.750 |
| Rhodophyta | 1 | 1 | 0 | 1.000 |
| Tracheophyta | 3 | 2 | 1 | 0.667 |
