# Supplementary material for: Recent extinctions of plant and animal genera are rare, localized, and decelerated
Source: PLoS Biol. 2025 Sep 4;23(9):e3003356. doi: 10.1371/journal.pbio.3003356 (PMC12410804; doi:10.1371/journal.pbio.3003356)
Supplement: S8 Table — (DOCX) [file pbio.3003356.s008.docx]

**S8 Table.** Distribution of extinct and possibly extinct genera among major groups of animals and plants. For each group, we give the combined number of extinct and possibly extinct genera in that group (EPE genera), the total number of genera assessed by IUCN (Assd.), the proportion of extinct and possibly extinct genera (among those assessed; EPE/Assd.), the number of genera assessed in the group relative to the total number of genera in the group, and the total number of genera in the group. Full data are in Dataset S1 (extinct) and Dataset S4 (possibly extinct).

| Taxon | EPE genera | Assd. | EPE/  Assd. | Assd./Total | Total genera |
| --- | --- | --- | --- | --- | --- |
| All | 139 | 22,760 | 0.0061 | 0.1087 | 209,312 |
| Animalia | 123 | 15,478 | 0.0080 | 0.0940 | 164,622 |
| Arthropoda | 22 | 3482 | 0.0063 | 0.0295 | 117,978 |
| Arachnida | 9 | 350 | 0.0257 | 0.0364 | 9609 |
| Diplopoda | 1 | 48 | 0.0208 | 0.0132 | 3645 |
| Ostracoda | 1 | 11 | 0.0909 | 0.0030 | 3645 |
| Insecta | 11 | 2532 | 0.0043 | 0.0284 | 89,311 |
| Chordata | 79 | 9998 | 0.0079 | 0.9254 | 10,804 |
| Actinopterygia | 11 | 4276 | 0.0026 | 0.8586 | 4980 |
| Amphibians | 2 | 558 | 0.0036 | 1.0072 | 554 |
| Birds | 38 | 2396 | 0.0159 | 1.0381 | 2308 |
| Mammals | 24 | 1308 | 0.0183 | 0.9864 | 1326 |
| Squamates | 3 | 1122 | 0.0027 | 0.9723 | 1154 |
| Turtles | 1 | 92 | 0.0109 | 0.9583 | 96 |
| Mollusks | 22 | 1698 | 0.0130 | 0.1042 | 16,294 |
| Bivalves | 2 | 183 | 0.0109 | 0.0562 | 3255 |
| Gastropods | 20 | 1345 | 0.0149 | 0.1378 | 9762 |
| Plantae | 16 | 6939 | 0.0023 | 0.3233 | 21,466 |
| Bryophyta | 3 | 119 | 0.0252 | 0.1137 | 1047 |
| Rhodophyta | 1 | 46 | 0.0217 | 0.0439 | 1235 |
| Tracheophyta | 12 | 6702 | 0.0018 | 0.4015 | 16,694 |
