# Supplementary material for: Recent extinctions of plant and animal genera are rare, localized, and decelerated
Source: PLoS Biol. 2025 Sep 4;23(9):e3003356. doi: 10.1371/journal.pbio.3003356 (PMC12410804; doi:10.1371/journal.pbio.3003356)
Supplement: S9 Table — (DOCX) [file pbio.3003356.s009.docx]

**S9 Table.** The proportion of extinct and possibly extinct (EPE) genera in each taxonomic group that are island endemics. Full data are in Dataset S1 (extinct) and Dataset S4 (possibly extinct).

| Taxon | EPE genera | Island | Mainland | Proportion island |
| --- | --- | --- | --- | --- |
| All | 139 | 97 | 42 | 0.698 |
| Animalia | 123 | 88 | 35 | 0.715 |
| Arthropoda | 22 | 19 | 3 | 0.864 |
| Arachnida | 9 | 9 | 0 | 1.000 |
| Diploda | 1 | 1 | 0 | 1.000 |
| Ostracoda | 1 | 0 | 1 | 0.000 |
| Insecta | 11 | 9 | 2 | 0.818 |
| Chordata | 79 | 55 | 24 | 0.696 |
| Actinopterygians | 11 | 2 | 9 | 0.182 |
| Amphibians | 2 | 0 | 2 | 0.000 |
| Birds | 38 | 33 | 5 | 0.868 |
| Mammals | 24 | 16 | 8 | 0.667 |
| Squamates | 3 | 3 | 0 | 1.000 |
| Turtles | 1 | 1 | 0 | 1.000 |
| Mollusks | 22 | 14 | 8 | 0.636 |
| Bivalves | 2 | 0 | 2 | 0.000 |
| Gastropods | 20 | 13 | 7 | 0.650 |
| Plantae | 16 | 9 | 9 | 0.563 |
| Bryophyta | 3 | 1 | 2 | 0.333 |
| Rhodophyla | 1 | 0 | 1 | 0.000 |
| Tracheophyta | 12 | 8 | 4 | 0.667 |
