# Supplementary material for: Recent extinctions of plant and animal genera are rare, localized, and decelerated
Source: PLoS Biol. 2025 Sep 4;23(9):e3003356. doi: 10.1371/journal.pbio.3003356 (PMC12410804; doi:10.1371/journal.pbio.3003356)
Supplement: S10 Table — (DOCX) [file pbio.3003356.s010.docx]

**S10 Table.** The ratio of species to genera among major groups of animals and plants. As in Table S1, for each group, we give the number of extinct genera in that group (Ext. genera), the total number of genera assessed by IUCN (Assd.), the proportion of extinct genera (among those assessed; Ext./Assd.), and the total number of genera and species in the group. Here, we also give the ratio of species to genera (Species/Genera). Among the thoroughly assessed groups of tetrapods (>95% assessed), there is a strong, negative relationship between the proportion of assessed genera that are extinct and the number of species per genus (*r*^2^=0.78; *P*=0.0487; *n*=5). However, we acknowledge that this relationship is no longer significant if all vertebrates are included (*r*^2^=0.52; *P*=0.1035; *n*=6; adding Actinopterygia) or if all relevant animal classes are included (*r*^2^=0.18; *P*=0.1919, *n*=11; 6 vertebrate clades, 3 arthropod classes, 2 mollusk classes).

| Taxon | Ext. genera | Assd. | Ext./  Assd. | Total genera | Total species | Species/Genera |
| --- | --- | --- | --- | --- | --- | --- |
| All | 102 | 22,760 | 0.0045 | 209,312 | 2,170,160 | 10.3681 |
| Animalia | 90 | 15,478 | 0.0059 | 164,622 | 1,553,708 | 9.4380 |
| Arthropoda | 11 | 3482 | 0.0032 | 117,978 | 1,204,321 | 10.2080 |
| Arachnida | 6 | 350 | 0.0171 | 9609 | 95,970 | 9.9875 |
| Ostracoda | 1 | 11 | 0.0909 | 3645 | 17,050 | 4.6776 |
| Insecta | 4 | 2532 | 0.0016 | 89,311 | 995,088 | 11.1418 |
| Chordata | 66 | 9998 | 0.0066 | 10,804 | 74,220 | 6.8697 |
| Actinopterygia | 4 | 4276 | 0.0009 | 4980 | 32,513 | 6.5287 |
| Amphibians | 1 | 558 | 0.0018 | 554 | 8,054 | 14.5379 |
| Birds | 37 | 2396 | 0.0154 | 2308 | 10,677 | 4.6261 |
| Mammals | 21 | 1308 | 0.0161 | 1326 | 6234 | 4.7014 |
| Squamates | 2 | 1122 | 0.0018 | 1154 | 11,769 | 10.1984 |
| Turtles | 1 | 92 | 0.0109 | 96 | 365 | 3.8021 |
| Mollusks | 13 | 1699 | 0.0082 | 16,294 | 138,354 | 8.4911 |
| Bivalves | 1 | 183 | 0.0055 | 3255 | 23,883 | 7.3373 |
| Gastropods | 12 | 1346 | 0.0097 | 9762 | 100,229 | 10.2672 |
| Plantae | 12 | 6939 | 0.0017 | 21,466 | 385,797 | 17.9725 |
| Bryophyta | 3 | 119 | 0.0252 | 1047 | 12,243 | 11.6934 |
| Tracheophyta | 9 | 6702 | 0.0013 | 16,694 | 365,207 | 21.8765 |
