# Supplementary material for: Recent extinctions of plant and animal genera are rare, localized, and decelerated
Source: PLoS Biol. 2025 Sep 4;23(9):e3003356. doi: 10.1371/journal.pbio.3003356 (PMC12410804; doi:10.1371/journal.pbio.3003356)
Supplement: S11 Table — (DOCX) [file pbio.3003356.s011.docx]

**S11 Table.** Relationships between extinction frequencies and genus-to-species ratios.

We tested relationships among the 11 animal classes with one or more extinct genera, and among vertebrate classes and tetrapod classes. We used both ordinary least-squares regression (OLS) and phylogenetic generalized least squares regression (PGLS). For relationships across animals, we used three phylogenies (Trees 1–3), which gave similar results. Note that for tetrapods, the maximum likelihood estimation of lambda failed. We therefore input lambda values of 1.00, 0.500, and 0.01. All three yielded similar results.

| Clade | Method | n | r^2^ | P | Estimate |
| --- | --- | --- | --- | --- | --- |
| Animalia | OLS | 11 | 0.18 | 0.1919 | -0.0032 |
|  | PGLS |  |  |  |  |
|  | Tree 1 | 11 | 0.25 | 0.1180 | -0.0030 |
|  | Tree 2 | 11 | 0.26 | 0.1118 | -0.0030 |
|  | Tree 3 | 11 | 0.26 | 0.1123 | -0.0030 |
| Vertebrata | OLS | 6 | 0.52 | 0.1035 | -0.0012 |
|  | PGLS | 6 | 0.58 | 0.0782 | -0.0013 |
| Tetrapoda | OLS | 5 | 0.78 | 0.0487 | -0.0013 |
|  | PGLS | 5 | 0.78 | 0.0484 | -0.0014 |
