# Supplementary material for: Recent extinctions of plant and animal genera are rare, localized, and decelerated
Source: PLoS Biol. 2025 Sep 4;23(9):e3003356. doi: 10.1371/journal.pbio.3003356 (PMC12410804; doi:10.1371/journal.pbio.3003356)
Supplement: S1 Fig — (DOCX) [file pbio.3003356.s012.docx]

**S1 Fig.** **Patterns of genus-level extinctions over time among centuries for possibly extinct genera.** Each column shows the number of genus-level extinctions in that century. We show patterns only for those groups with the most genus-level extinctions. Data for all groups are given in Dataset S4 (available on figshare at: doi:10.6084/m9.figshare.27377613). We excluded the 2000s given that this century is not fully comparable to others (only 24% elapsed, with potential bias against reporting of the most recent extinctions).
