# Supplementary material for: Recent extinctions of plant and animal genera are rare, localized, and decelerated
Source: PLoS Biol. 2025 Sep 4;23(9):e3003356. doi: 10.1371/journal.pbio.3003356 (PMC12410804; doi:10.1371/journal.pbio.3003356)
Supplement: S4 Fig — (DOCX) [file pbio.3003356.s015.docx]

**S4 Fig.** **Patterns of genus-level extinctions over time among decades for the combined sets of extinct and "possibly extinct" genera.** Each column shows the number of genus-level extinctions in that decade. We show patterns only for those groups with the most genus-level extinctions, and only from the 1800s to 2010s. There were relatively few extinctions before the 1800s in most groups, except birds and mammals. Data for all groups are given in Dataset S5 (available on figshare at: doi:10.6084/m9.figshare.27377613).
